# High-density EEG network analysis in MCI: an exploratory study of electrode density and cognitive performance

**DOI:** 10.3389/fnagi.2026.1854225

**Published:** 2026-06-10

**Authors:** Serena Dattola, Augusto Ielo, Viviana Lo Buono, Salvatore Bertino, Simona De Salvo, Angelo Quartarone, Lilla Bonanno

**Affiliations:** Istituto di Ricovero e Cura a Carattere Scientifico (IRCCS) Centro Neurolesi Bonino-Pulejo, Messina, Italy

**Keywords:** Alzheimer's disease, electrode density, functional connectivity, graph theory, high-density electroencephalography, mild cognitive impairment, montreal cognitive assessment, source-level analysis

## Abstract

**Introduction:**

Electroencephalography-derived functional connectivity and graph-theoretical measures are increasingly investigated as markers of cognitive dysfunction in mild cognitive impairment, but the extent to which their clinical sensitivity depends on electrode density remains unclear. This study examined whether source-level network metrics are associated with cognitive performance and whether electrode density influences their ability to capture and classify cognitive differences within a mild cognitive impairment cohort.

**Methods:**

Resting-state high-density electroencephalography was acquired from 17 individuals with mild cognitive impairment. From the original recordings, three montages were derived (173, 64, and 18 channels). Source-level lagged linear connectivity was reconstructed with exact low-resolution electromagnetic tomography across 84 cortical regions and used to compute graph-theoretical metrics of network integration and segregation. Associations with Montreal Cognitive Assessment scores were examined using Spearman correlations, between-group differences were assessed after stratification by cognitive performance, and single-feature binary classification was evaluated with leave-one-out cross-validation.

**Results:**

Correlation patterns appeared progressively clearer with increasing electrode density, especially in the alpha and beta bands, although none survived correction for multiple comparisons. In contrast, the 173-channel montage showed the strongest and most consistent between-group differences, with several metrics remaining significant after false discovery rate correction. The same configuration also achieved the best classification performance, with balanced accuracy up to 0.82 and area under the curve up to 0.94.

**Discussion:**

These findings suggest that electrode density may affect the sensitivity of source-level electroencephalography network analysis to cognition-related alterations. In this exploratory sample, high-density recordings showed the clearest between-group differences and the best classification performance, consistent with the possibility that denser montages provide richer network information within the mild cognitive impairment spectrum.

## 1 Introduction

Alzheimer's disease (AD) is a progressive neurodegenerative disorder and the leading cause of dementia worldwide (Scheltens et al., 2021). The pathological process typically develops gradually, often preceded by a prodromal phase characterized by mild cognitive impairment (MCI), in which subtle cognitive deficits, particularly affecting memory, are present but daily functional independence is largely preserved. As the disease progresses, impairments extend to language, orientation, executive functioning, and behavior, ultimately leading to severe cognitive decline and loss of autonomy. Early identification of individuals at risk remains challenging, since initial symptoms may overlap with normal aging processes (Rossini et al., 2020).

From a clinical perspective, cognitive screening tools remain essential for identifying individuals with possible cognitive impairment. The Montreal Cognitive Assessment (MoCA) is a sensitive instrument for detecting mild cognitive deficits, particularly in early Alzheimer's disease and MCI populations (Nasreddine et al., 2005). Compared with traditional screening tools such as the Mini-Mental State Examination (MMSE), the MoCA shows greater sensitivity to executive and memory dysfunction, and is increasingly used both in clinical practice and research settings (Pinto et al., 2019; Ciesielska et al., 2016).

Electroencephalography (EEG) has long been used as a non-invasive method to investigate brain function in AD (Bi et al., 2025). It offers high temporal resolution, relatively low cost, and ease of application compared with other neuroimaging techniques. EEG signals reflect the synchronous activity of large populations of cortical pyramidal neurons recorded through electrodes placed on the scalp. However, scalp-recorded potentials result from the superposition of multiple neural sources filtered by head tissues, which limits spatial resolution and requires appropriate head modeling and signal processing approaches (Wang and Ren, 2013).

In recent years, the development of high-density EEG (HD-EEG) systems, incorporating up to 256 electrodes, has improved spatial sampling of scalp potentials, although the optimal electrode configuration for clinical applications remains an open question (La Foresta et al., 2019; Stoyell et al., 2021; Song et al., 2015; Tucker, 1993).

Functional connectivity describes the statistical dependencies between neurophysiological signals recorded from spatially distributed brain regions, reflecting coordinated neural activity across large-scale networks (Bastos and Schoffelen, 2016). Importantly, the estimation of EEG functional connectivity at the sensor level is affected by volume conduction and field spread, which may introduce spurious correlations between neighboring electrodes. Source-level connectivity analyses, obtained after cortical source reconstruction, have been shown to reduce these confounding effects and provide a more accurate representation of interactions between distinct brain regions (Michel and He, 2019). Among source localization methods, low resolution electromagnetic tomography (LORETA) and its variants, such as eLORETA, are widely used due to their stability and ability to estimate distributed cortical activity without localization bias (Pascual-Marqui, 2007a). The developers of LORETA later introduced the lagged linear connectivity method to estimate functional connectivity at the cortical source level (Pascual-Marqui, 2007b; Pascual-Marqui et al., 2011). This approach has been widely applied in studies investigating brain network alterations in patients with MCI and AD (Paitel et al., 2025; Dattola et al., 2021; La Foresta et al., 2019).

Graph theoretical analysis provides a powerful tool for characterizing brain networks derived from EEG functional connectivity. Within this framework, brain regions are modeled as nodes interconnected by edges representing functional interactions (Rubinov and Sporns, 2010). Quantitative metrics such as clustering coefficient and characteristic path length, capture complementary aspects of network segregation and integration, respectively (Rubinov and Sporns, 2010). Healthy brain networks typically exhibit small-world properties, combining efficient global communication with local specialization (Rubinov and Sporns, 2010; Bullmore and Sporns, 2009; Bassett and Bullmore, 2006). Disruptions of this balance have been consistently reported in both AD and MCI, often reflecting decreased connectivity, reduced network efficiency, and altered oscillatory synchronization (Wang et al., 2014; Stam et al., 2007; König et al., 2005).

Over the years, several studies have investigated the relationship between EEG functional connectivity and the progression of MCI and AD (Paitel et al., 2025; Cassani et al., 2018). However, to our knowledge, only a limited number of studies have examined the association between EEG functional connectivity measures and cognitive status as assessed by MoCA. A significant positive correlation between MoCA scores and the clustering coefficient, together with a negative correlation with characteristic path length in the lower alpha band, has been reported in patients with amnestic mild cognitive impairment (aMCI) with type 2 diabetes (Zeng et al., 2015). In another study, the α-band phase lag index has been shown to be positively correlated with MoCA scores in patients with MCI with type 2 diabetes (Kuang et al., 2022). However, the extent to which EEG connectivity metrics reflect cognitive performance may also depend on methodological factors, including the spatial sampling of scalp recordings. In particular, the impact of electrode density on the ability of connectivity-based network measures to capture clinically relevant cognitive alterations remains largely unexplored.

The present cross-sectional exploratory study addresses this issue by investigating the relationship between EEG functional connectivity and MoCA in individuals with MCI using HD-EEG recordings acquired with a 256-channel system. Starting from these recordings, three electrode configurations were derived to simulate high-, medium-, and low-density EEG montages, allowing us to systematically evaluate the impact of electrode density on connectivity-based network measures. Functional connectivity was reconstructed at the cortical source level using lagged linear connectivity, and graph-theoretical metrics were computed to characterize the resulting brain networks. Specifically, this study investigates two main aspects. First, we evaluate the correlation between MoCA scores and EEG connectivity-derived graph metrics across the three electrode density configurations, in order to determine whether these measures reflect variations in cognitive performance. Second, we assess whether these connectivity features can discriminate subjects according to their MoCA-defined cognitive status across the same electrode configurations using a single-feature classification framework.

## 2 Materials and methods

### 2.1 EEG acquisition and preprocessing

The dataset consists of HD-EEG recordings acquired at the IRCCS Centro Neurolesi Bonino-Pulejo in Messina, Italy. The study was conducted according to a protocol approved by the local Ethics Committee (N. CEL/E108/25 dated 22.10.2025). Written informed consent was obtained from all participants or their caregivers.

**Figure 1:**
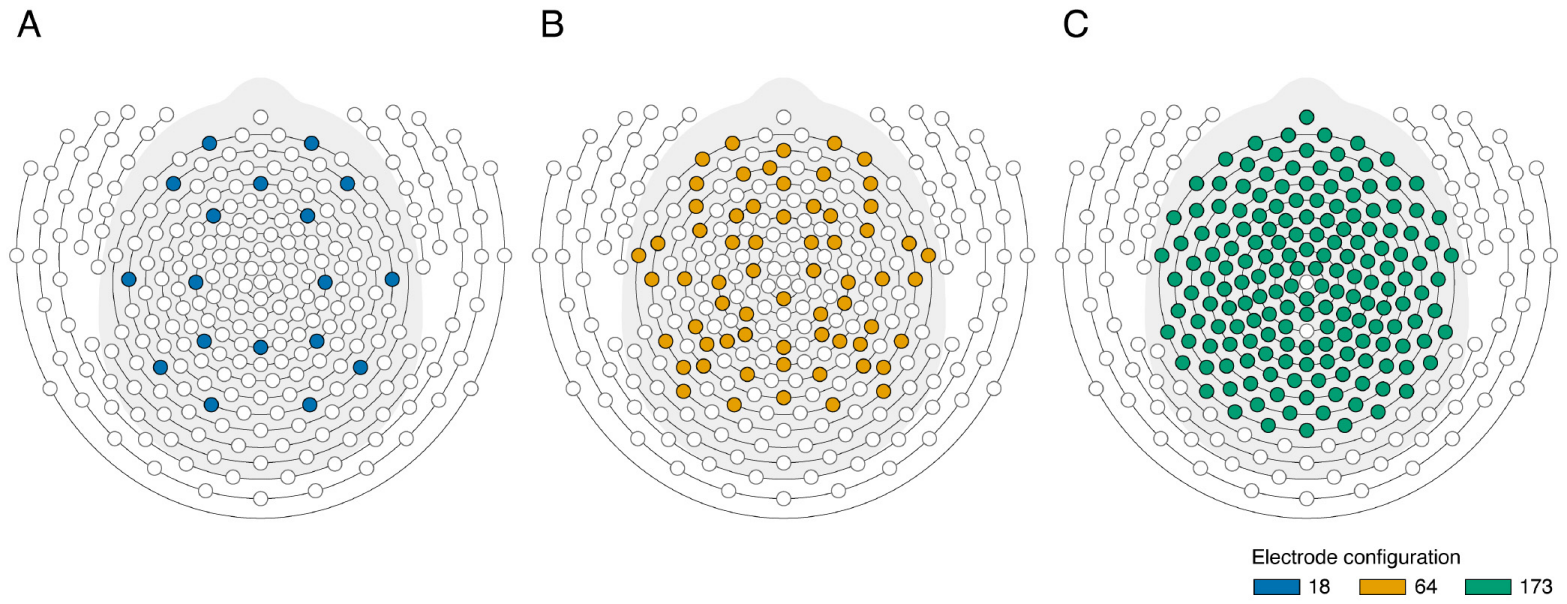
Electrode configurations used in the study. Schematic representation of the three EEG montages derived from the original 256-channel HydroCel Geodesic Sensor Net. Panel **(A)** shows the low-density 18-channel configuration (blue), panel **(B)** the medium-density 64-channel configuration (orange), and panel **(C)** the high-density 173-channel configuration (green). White circles indicate electrodes from the original montage that were not included in the corresponding derived configuration.

EEG recordings were obtained during eyes-closed resting-state conditions from 17 subjects evaluated in a real-world clinical setting for early cognitive impairment within the MCI spectrum (mean age: 77 ± 7.4 years; males = 5; education range: 8–18 years; disease duration range: 1–4 years). Demographic and clinical data are reported in Table 1. MCI was diagnosed according to the Mayo Clinic criteria originally proposed by Petersen (2004), requiring subjective cognitive complaints, objective impairment on neuropsychological testing, preserved general cognitive function, intact or minimally impaired functional activities of daily living, and absence of dementia. The diagnostic framework was also considered in line with the recommendations of the National Institute on Aging-Alzheimer's Association (NIA-AA) criteria (Albert et al., 2011). All participants underwent neurological and cognitive assessment, including the MoCA, as well as EEG recording. Cranial CT was available for all subjects, whereas brain MRI was not available for any participant. Based on the available clinical and instrumental information, the cohort was clinically heterogeneous and included different clinical presentations within the MCI spectrum, including cases with a vascular component. All patients received donepezil treatment. Subjects with clinically evident depression, sleep disorders, or epilepsy were not included. A detailed and uniform subtype classification, such as amnestic vs. non-amnestic MCI or single-domain vs. multidomain MCI, was not consistently available for all participants and was therefore not used in the present analyses. EEG signals were recorded using a 256-channel HydroCel Geodesic Sensor Net, part of the Geodesic EEG System (GES). In this system, the distance between electrode pairs follows geodesic lines, representing the shortest path between two points on the surface of a sphere. This geodesic tessellation of the scalp allows accurate and uniform sampling of the scalp electrical field (Electrical Geodesics, Inc.). Electrode impedance was maintained below 50 kΩ, in accordance with the manufacturer's guidelines. The reference electrode was Cz, located at the vertex of the scalp. Signals were sampled at 250 Hz and band-pass filtered between 1 and 40 Hz. Filtering was applied to the original 256-channel recordings before deriving the reduced montages. No independent component analysis (ICA), channel interpolation, or automated artifact-rejection procedures were applied. Artifact detection was based on visual inspection, and segments contaminated by ocular, muscular, movement-related, or other non-physiological artifacts were excluded from further analysis. After filtering, electrodes located on the face and neck were excluded because of excessive noise, resulting in the 173-channel configuration. The 64-channel (medium-density) configuration was then derived as a fixed subset of the 173-channel montage based on the 10-10 electrode position equivalence for the HydroCel GSN system (Luu and Ferree, 2005), and the 18-channel (low-density) configuration was obtained as a further fixed subset of the 64-channel montage. Thus, the three electrode configurations were nested and derived from the same filtered recordings. The three montages considered in the present study are illustrated in Figure 1, while the full correspondence between the 173-, 64-, and 18-channel configurations and the original 256-channel HydroCel layout is reported in Supplementary Table S1.

**Table 1:** Demographic and clinical data of the overall sample and the two MoCA-defined groups.

Variable	All	Group 1	Group 2
	*n* = 17	*n* = 8	*n* = 9
Age, years (mean ± SD)	77 ± 7.40	78.38 ± 2.07	76.33 ± 10.15
Female, *n* (%)	12 (70.6)	6 (75.0)	6 (66.7)
Male, *n* (%)	5 (29.4)	2 (25.0)	3 (33.3)
Education range (years)	8–18	8–16	10–18
Disease duration range (years)	1–4	2–4	1–3
MoCA (mean ± SD)	22.06 ± 4.29	18.63 ± 3.85	25.11 ± 1.27
MoCA range	12–28	12–23	24–28

Group 1: MoCA < 24; Group 2: MoCA ≥24.

Finally, common average rereferencing was performed separately for each derived montage (173, 64, and 18 channels), so that each configuration was treated as an independent electrode set for subsequent analyses.

### 2.2 Functional connectivity and graph measures

Brain functional connectivity was estimated at the cortical source level using the eLORETA algorithm, as implemented in the LORETA-KEY software (v20240713) (The KEY Institute for Brain-Mind Research, University of Zurich, Zurich, Switzerland). LORETA is a widely used method for estimating the cortical generators of scalp-recorded EEG activity. Pascual-Marqui (2007b) and Pascual-Marqui et al. (2011) later introduced a framework for assessing functional connectivity based on this source reconstruction approach. In this framework, overall connectivity is decomposed into instantaneous and lagged components: the instantaneous component mainly reflects zero-lag correlations arising from volume conduction and common sources, whereas the lagged component captures delayed interactions that are more likely to represent genuine physiological connectivity.

Functional connectivity was computed using the Connectivity Toolbox available in the LORETA-KEY software. Specifically, we calculated lagged linear connectivity (LLC), which quantifies the statistical dependence between reconstructed cortical sources. LLC was computed for each pair of regions of interest (ROIs) across the cortex. The analysis included 84 cortical ROIs provided by the LORETA-KEY atlas, corresponding to Brodmann areas (42 per hemisphere). Source reconstruction was performed using the eLORETA implementation with the same reconstruction settings across all electrode configurations. In particular, the same head model, cortical solution space, ROIs, and frequency-band definitions were used for the 18-, 64-, and 173-channel montages; the only varying factor was the number of input electrodes. According to the LORETA-KEY implementation, the inverse solution relies on an electric potential model computed with the boundary element method on the MNI152 template, and the cortical solution space consists of 6,239 cortical grey-matter voxels at 5-mm resolution. Therefore, source reconstruction was based on a template head model. Electrode positions were defined according to the standard HydroCel Geodesic Sensor Net layout. Connectivity was estimated for the three electrode montages described in Section 2.1 and for the following frequency bands: delta (1–4 Hz), theta (4–8 Hz), alpha 1 (8–10.5 Hz), alpha 2 (10.5–13 Hz), beta 1 (13–18 Hz), beta 2 (18–30 Hz).

For each participant, 4 min of artifact-free EEG data were selected for analysis. The signals were segmented into non-overlapping epochs of 250 samples (1 s) (Nunez et al., 1997). LLC was computed using windows of three consecutive epochs, resulting in 80 connectivity matrices per subject. These windows were non-overlapping and were used to characterize within-subject temporal variability.

Graph-theoretical measures were then extracted from the ROI-by-ROI connectivity matrices using the Brain Connectivity Toolbox implemented in MATLAB. For each subject, graph-theoretical parameters were computed for all 80 connectivity matrices and then averaged, so that the final statistical analyses were performed on one mean value per subject for each metric, frequency band, and electrode configuration. We selected a set of complementary network indices that are widely used to characterize both integration and segregation properties of weighted brain networks (La Foresta et al., 2019; Rubinov and Sporns, 2010). In particular, global efficiency and characteristic path length were used to quantify network integration, while the mean clustering coefficient and transitivity were used to describe network segregation. Global efficiency is defined as the average inverse shortest path length in the network and reflects how efficiently information can be exchanged at the whole-network level, whereas characteristic path length corresponds to the average shortest path length and provides a complementary estimate of global communication distance. The clustering coefficient quantifies the fraction of triangles around a node, that is, the extent to which its neighbors are also interconnected. In the present study, the mean clustering coefficient was computed by averaging this quantity across nodes. Transitivity, defined as the ratio of triangles to triplets in the network, was included as a global measure of clustering organization. In addition, mean nodal strength was computed as the average across nodes of the sum of weights of all links connected to each node, providing a summary measure of the overall weight of functional connections across the network.

### 2.3 Statistical analysis

Associations between global cognitive status, as indexed by MoCA, and EEG graph-theoretical metrics were first examined separately within each channel configuration (18, 64, and 173 channels). Given the limited sample size and the non-parametric analytical framework, Spearman's rank correlation coefficient (ρ) was used to assess the relationship between MoCA scores and each EEG metric. Corresponding *p*-values were adjusted for multiple comparisons within each channel configuration using the Benjamini–Hochberg false discovery rate (FDR) procedure. For visualization purposes, approximate 95% confidence intervals for correlation coefficients were estimated using Fisher's *z* transformation. Subsequently, to further characterize between-group differences, the sample within each channel configuration was stratified into two subgroups according to cognitive performance, defined as MoCA < 24 (group 1) and MoCA ≥24 (group 2). Although a MoCA cutoff of 24 has been widely used to distinguish cognitively impaired individuals from healthy controls in previous studies (Carson et al., 2018), in the present study all participants were clinically diagnosed with MCI. Therefore, this threshold was not interpreted diagnostically, but was used to stratify the cohort into subgroups with relatively lower and higher cognitive performance. Graph-theoretical EEG metrics were then compared between groups using the Mann–Whitney *U*-test, consistent with the non-parametric analytical approach. Descriptive statistics are reported as median and interquartile range (IQR). The magnitude of between-group differences was quantified using the rank-based effect size *r*. *P*-values were adjusted within each channel configuration using the Benjamini–Hochberg FDR procedure. Statistical analyses were conducted in R (version 4.4.2) (R Foundation for Statistical Computing, Vienna, Austria). Given the limited sample size, the present analyses were intended as exploratory.

### 2.4 Single-feature binary classification

To further assess the potential clinical relevance of EEG-derived graph measures, a single-feature binary classification analysis was performed to distinguish between lower and higher cognitive performance (groups 1 and 2). For each electrode configuration (18, 64, and 173 channels), each graph metric, and each frequency band, classification performance was evaluated independently using a single predictor at a time. For every combination of electrode configuration, metric, and frequency band, a logistic regression classifier with ridge regularization was trained using the corresponding feature value as the only input variable. Regularization was implemented with a ridge penalty parameter set to λ = 10^−3^. Classification performance was estimated using leave-one-out cross-validation (LOOCV), so that each subject was iteratively used once as the test case while the remaining subjects constituted the training set. All classification analyses were performed at the subject level, with each model trained independently on a single predefined feature and without any supervised feature selection before cross-validation. To avoid information leakage, feature standardization was performed within each cross-validation fold using the mean and standard deviation estimated from the training data only, and the same parameters were then applied to the held-out test subject.

For each model, predicted probabilities for the positive class (MoCA < 24) were collected across all LOOCV folds. Classification performance was summarized using balanced accuracy as the primary metric, together with accuracy, sensitivity, specificity, and the area under the receiver operating characteristic curve (AUC). Balanced accuracy was selected as the main summary measure because it gives equal weight to sensitivity and specificity and is therefore less influenced by small differences in class size. To facilitate interpretation and comparison across electrode densities, for each combination of electrode configuration and graph-theoretical metric, the frequency band associated with the highest balanced accuracy was retained for presentation in the main results table. When multiple bands showed the same balanced accuracy, the band with the highest AUC was selected.

To report the classification results, one best-performing model was identified for each electrode configuration and visualized by means of a confusion matrix and ROC curve. In addition, for the overall best-performing model, the statistical significance of the observed balanced accuracy was assessed by permutation testing. Class labels were randomly reassigned while preserving the original class sizes, and the full LOOCV procedure was repeated for each unique permuted labeling sampled without replacement. This procedure generated an empirical null distribution of balanced accuracy values, from which a one-sided p-value was computed as the proportion of permuted balanced accuracy values greater than or equal to the observed one.

A supplementary sensitivity analysis was performed to examine the effect of the MoCA cutoff used to define the binary outcome. In addition to the main threshold, the same single-feature classification procedure was repeated using adjacent thresholds at MoCA < 23 and MoCA < 25. For each alternative cutoff, subjects were reclassified accordingly, and the same logistic ridge classifier, leave-one-out cross-validation scheme, and performance measures were applied. For the best-performing model identified under each threshold, statistical significance of the observed balanced accuracy was further assessed by permutation testing, using class-label reassignment while preserving the corresponding class sizes.

## 3 Results

### 3.1 Sample characteristics

A total of 51 EEG recordings were analyzed, corresponding to 17 subjects evaluated under each of the three electrode configurations (18, 64, and 173 channels). Demographic and clinical data of the two MoCA-defined groups are reported in Table 1. Group 1 (MoCA < 24) included 8 subjects, whereas group 2 (MoCA ≥24) included nine subjects.

### 3.2 Associations between MoCA and EEG network metrics

**Figure 2:**
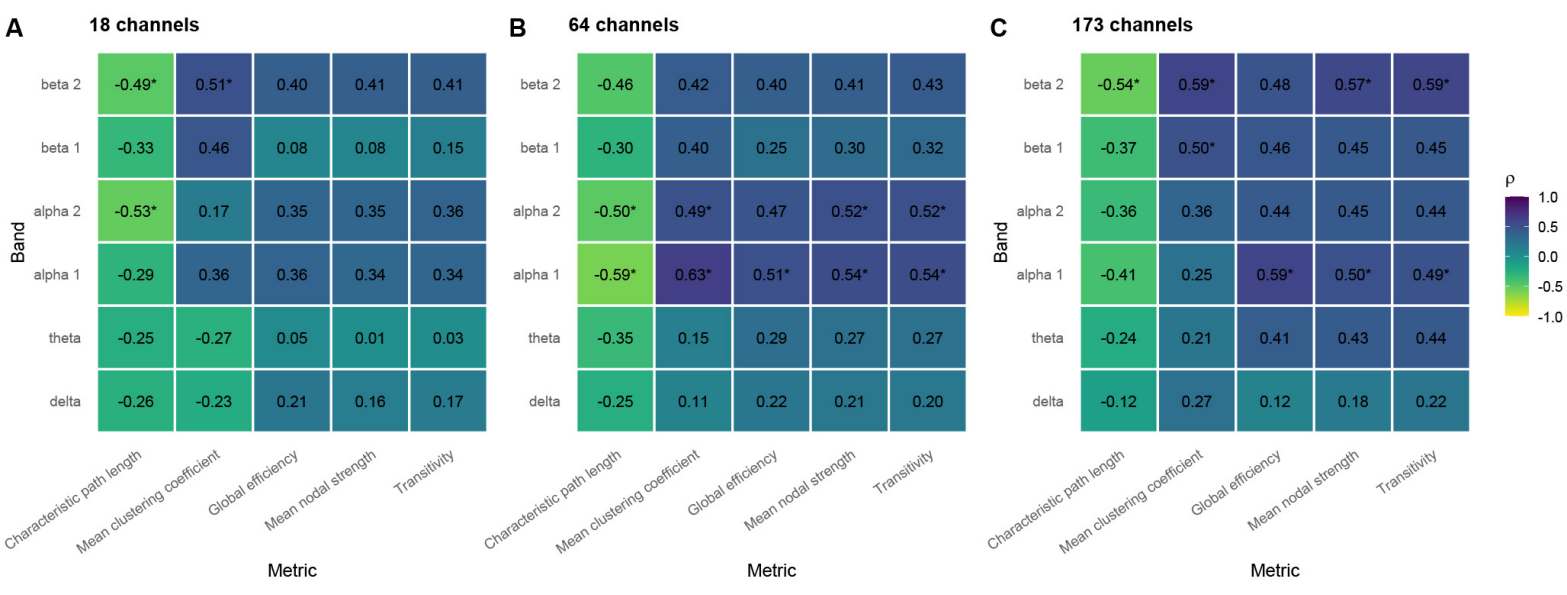
Correlations between MoCA scores and EEG graph-theoretical metrics across channel configurations. Heatmaps show Spearman's correlation coefficients between MoCA and EEG network metrics for the 18-channel **(A)**, 64-channel **(B)**, and 173-channel **(C)** configurations. Rows represent frequency bands, columns represent graph metrics, and numerical values within each cell indicate the correlation coefficient. One asterisk (*) denotes *p* < 0.05.

Associations between MoCA scores and EEG graph-theoretical metrics are reported in Table 2 and visually summarized in Figures 2A–C.

**Table 2:** Correlations between MoCA and EEG network metrics across channel configurations.

Metric	Band	18 channels			64 channels			173 channels		
		ρ	*p*-value	*p*FDR	ρ	*p*-value	*p*FDR	ρ	*p*-value	*p*FDR
Characteristic path length	Delta	–0.26	0.31	0.52	–0.25	0.33	0.39	–0.12	0.64	0.64
	Theta	–0.25	0.33	0.52	–0.35	0.17	0.29	–0.24	0.36	0.43
	Alpha 1	–0.29	0.25	0.48	–0.59	**0.01**	0.14	–0.41	0.10	0.16
	Alpha 2	–0.53	**0.03**	0.38	–0.50	**0.04**	0.14	–0.36	0.16	0.21
	Beta 1	–0.33	0.19	0.38	–0.30	0.25	0.37	–0.37	0.14	0.21
	Beta 2	–0.49	0.05	0.38	–0.46	0.06	0.17	–0.54	**0.03**	0.15
Mean clustering coefficient	Delta	–0.23	0.37	0.55	0.11	0.67	0.67	0.27	0.30	0.39
	Theta	–0.27	0.29	0.51	0.15	0.57	0.59	0.21	0.41	0.46
	Alpha 1	0.36	0.16	0.38	0.63	**0.01**	0.14	0.25	0.33	0.42
	Alpha 2	0.17	0.51	0.67	0.49	**0.04**	0.14	0.36	0.16	0.21
	Beta 1	0.46	0.06	0.38	0.40	0.11	0.21	0.50	**0.04**	0.15
	Beta 2	0.51	**0.04**	0.38	0.42	0.09	0.21	0.59	**0.01**	0.12
Global efficiency	Delta	0.21	0.41	0.59	0.22	0.40	0.46	0.12	0.63	0.64
	Theta	0.05	0.84	0.90	0.29	0.27	0.38	0.41	0.10	0.16
	Alpha 1	0.36	0.15	0.38	0.51	**0.04**	0.14	0.59	**0.01**	0.12
	Alpha 2	0.35	0.17	0.38	0.47	0.05	0.16	0.44	0.08	0.15
	Beta 1	0.08	0.76	0.85	0.25	0.32	0.39	0.46	0.07	0.15
	Beta 2	0.40	0.11	0.38	0.40	0.11	0.21	0.48	0.05	0.15
Mean nodal strength	Delta	0.16	0.55	0.68	0.21	0.43	0.47	0.18	0.48	0.52
	Theta	0.01	0.97	0.97	0.27	0.30	0.39	0.43	0.09	0.15
	Alpha 1	0.34	0.19	0.38	0.54	**0.03**	0.14	0.50	**0.04**	0.15
	Alpha 2	0.35	0.17	0.38	0.52	**0.03**	0.14	0.45	0.07	0.15
	Beta 1	0.08	0.75	0.85	0.30	0.24	0.37	0.45	0.07	0.15
	Beta 2	0.41	0.10	0.38	0.41	0.11	0.21	0.57	**0.02**	0.12
Transitivity	Delta	0.17	0.52	0.67	0.20	0.43	0.47	0.22	0.39	0.45
	Theta	0.03	0.91	0.95	0.27	0.30	0.39	0.44	0.08	0.15
	Alpha 1	0.34	0.18	0.38	0.54	**0.03**	0.14	0.49	0.05	0.15
	Alpha 2	0.36	0.15	0.38	0.52	**0.03**	0.14	0.44	0.08	0.15
	Beta 1	0.15	0.56	0.68	0.32	0.22	0.36	0.45	0.07	0.15
	Beta 2	0.41	0.10	0.38	0.43	0.08	0.21	0.59	**0.01**	0.12

Spearman's rank correlation coefficients (ρ) are reported for each graph-theoretical metric across frequency bands and channel configurations. *p*FDR indicates Benjamini–Hochberg false discovery rate adjusted *p*-values. Bold values indicate *p*-values or *p*FDR values < 0.05.

#### 3.2.1 18-channel configuration

At the 18-channel configuration, correlations between cognitive performance and network metrics were generally weak and heterogeneous (Table 2; Figure 2A). Most associations were not statistically significant and did not survive correction for multiple comparisons. A moderate negative correlation was observed between MoCA and alpha 2 characteristic path length (ρ = −0.53, *p* = 0.03), suggesting that higher cognitive performance may be related to shorter path length in this band; however, this association did not remain significant after FDR correction (*p*_FDR_ = 0.38). In the beta 2 band, MoCA showed a negative correlation with characteristic path length (ρ = −0.49, *p* = 0.05) and a positive correlation with mean clustering coefficient (ρ = 0.51, *p* = 0.04), but neither survived FDR correction. Additional moderate positive associations were observed for beta 2 mean nodal strength and transitivity (ρ ≈ 0.41), although these did not reach statistical significance. Across delta, theta, alpha 1, and beta 1 bands, correlations were small and non-significant. None of the observed associations at the 18-channel level survived FDR correction.

#### 3.2.2 64-channel configuration

At the 64-channel configuration, a clearer and more coherent pattern of associations emerged, particularly within the alpha frequency range (Table 2; Figure 2B). In the alpha 1 band, MoCA showed associations with all network metrics, including characteristic path length (ρ = −0.59, *p* = 0.01), mean clustering coefficient (ρ = 0.63, *p* = 0.006), global efficiency (ρ = 0.51, *p* = 0.04), mean nodal strength (ρ = 0.54, *p* = 0.03), and transitivity (ρ = 0.54, *p* = 0.03), all indicating correlations of moderate magnitude. A similar profile was observed in the alpha 2 band, where MoCA showed associations with characteristic path length (ρ = −0.50, *p* = 0.04), mean clustering coefficient (ρ = 0.49, *p* = 0.04), mean nodal strength (ρ = 0.52, *p* = 0.03), and transitivity (ρ = 0.52, *p* = 0.03). Global efficiency also showed a similar trend (ρ = 0.47, *p* = 0.05). Correlations in theta and beta bands were generally weaker and not statistically significant. Although several associations showed moderate correlation magnitudes, none survived FDR correction (all *p*_FDR_ > 0.14).

#### 3.2.3 173-channel configuration

At the 173-channel configuration, correlations appeared stronger and more coherent across metrics, particularly in the alpha and beta bands (Table 2; Figure 2C). In the alpha 1 band, MoCA showed positive associations with global efficiency (ρ = 0.59, *p* = 0.01), mean nodal strength (ρ = 0.50, *p* = 0.04), and transitivity (ρ = 0.49, *p* = 0.046), although these associations did not survive FDR correction. In the beta 1 band, MoCA showed an association with mean clustering coefficient (ρ = 0.50, *p* = 0.04), again without remaining significant after correction. The strongest trend-level associations were observed in the beta 2 band, where MoCA showed a negative correlation with characteristic path length (ρ = −0.54, *p* = 0.025) and positive correlations with mean clustering coefficient (ρ = 0.59, *p* = 0.012), mean nodal strength (ρ = 0.57, *p* = 0.017), and transitivity (ρ = 0.59, *p* = 0.013). Additional moderate correlations were observed in the theta band, particularly for global efficiency, mean nodal strength, and transitivity (ρ≈0.41–0.44), although these did not reach statistical significance. None of the associations observed at the 173-channel level remained significant after FDR correction (all *p*_FDR_>0.12).

### 3.3 Between-group differences in EEG network metrics

**Figure 3:**
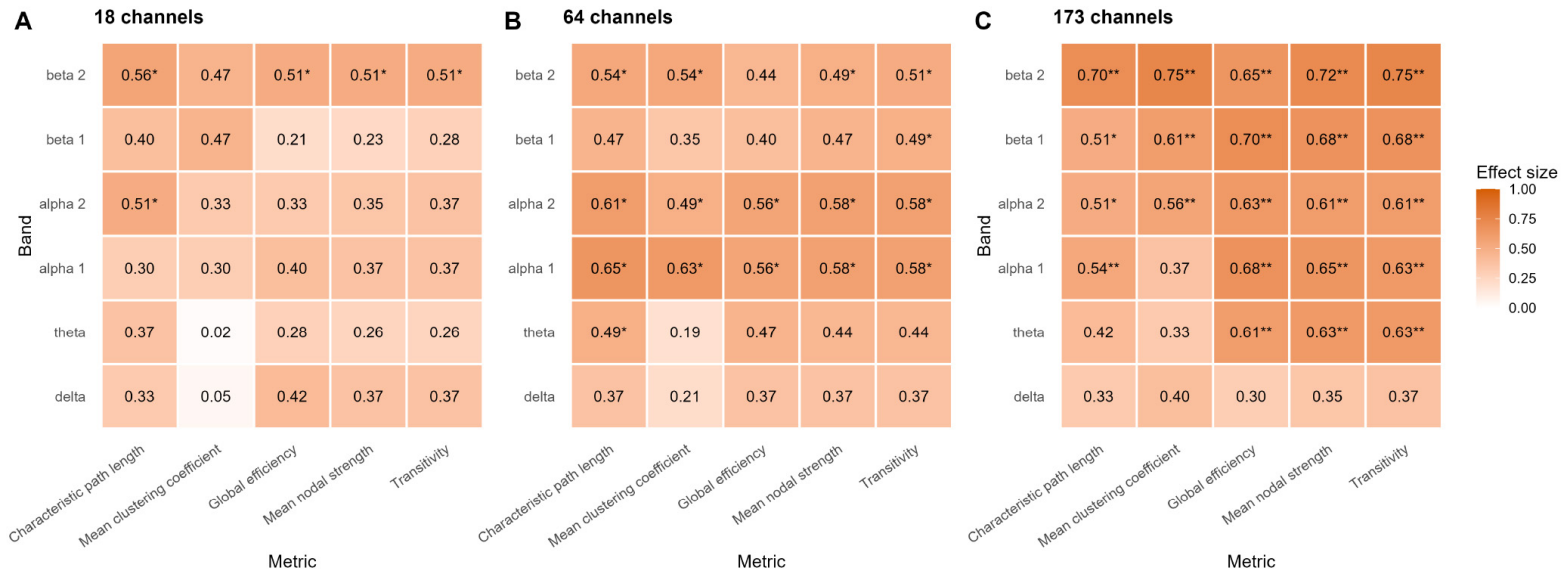
Between-group differences in EEG graph-theoretical metrics across channel configurations. Heatmaps show the effect sizes for comparisons between group 1 (MoCA < 24) and group 2 (MoCA ≥24) across frequency bands and graph metrics for the 18-channel **(A)**, 64-channel **(B)**, and 173-channel **(C)** configurations. Rows represent frequency bands, columns represent graph metrics, and numerical values within each cell indicate the effect size. One asterisk (*) denotes *p* < 0.05, whereas two asterisks (**) denote significance after FDR correction.

Group differences in EEG network metrics between individuals with lower (MoCA < 24) and higher (MoCA ≥24) cognitive performance are reported in Table 3 and illustrated in Figures 3A–C.

**Table 3:** (Continued)

Metric	Band	18ch					64ch					173ch				
		Group 1	Group 2	ES	*p*-value	*p*FDR	Group 1	Group 2	ES	*p*-value	*p*FDR	Group 1	Group 2	ES	*p*-value	*p*FDR
Characteristic path length	Delta	7.41 [6.27–8.36]	5.93 [5.34–6.44]	0.33	0.19	0.29	9.68 [8.51–16.15]	7.31 [6.08–9.04]	0.37	0.14	0.15	14.42 [12.80–17.45]	11.61 [10.90–13.68]	0.33	0.19	0.20	
	Theta	6.93 [6.54–7.48]	6.06 [5.68–6.55]	0.37	0.14	0.25	9.45 [8.41–13.31]	7.22 [6.82–7.98]	0.49	0.05	0.09	13.07 [12.30–16.57]	10.95 [9.96–11.46]	0.42	0.09	0.12	
	Alpha 1	5.05 [4.68–5.55]	4.70 [3.37–5.12]	0.30	0.23	0.31	6.18 [5.86–7.60]	5.01 [4.94–5.63]	0.65	0.01	0.08	9.41 [8.67–9.89]	7.89 [7.17–8.42]	0.54	0.03	0.05
	Alpha 2	4.08 [3.76–4.18]	3.61 [3.43–3.99]	0.51	0.04	0.23	5.00 [4.64–5.40]	4.41 [3.79–4.52]	0.61	0.01	0.08	7.17 [6.89–7.85]	5.95 [5.92–6.48]	0.51	0.04	0.05
	Beta 1	7.46 [7.00–8.11]	6.97 [6.71–7.20]	0.40	0.11	0.25	8.41 [7.97–9.06]	7.38 [6.92–7.89]	0.47	0.06	0.10	12.59 [11.89–13.14]	10.34 [10.04–10.65]	0.51	0.04	0.05
	Beta 2	20.42 [16.42–25.43]	15.27 [12.95–16.58]	0.56	0.02	0.23	17.98 [16.66–19.55]	15.57 [12.68–16.66]	0.54	0.03	0.08	28.41 [24.50–34.81]	21.18 [19.81–23.47]	0.70	<0.001	0.02
Mean clustering coefficient	Delta	0.24 [0.23–0.24]	0.24 [0.23–0.24]	0.05	0.89	0.92	0.15 [0.12–0.16]	0.15 [0.14–0.17]	0.21	0.41	0.43	0.09 [0.08–0.10]	0.10 [0.09–0.11]	0.40	0.11	0.14
	Theta	0.23 [0.23–0.24]	0.23 [0.22–0.24]	0.02	0.96	0.96	0.16 [0.14–0.16]	0.16 [0.15–0.17]	0.19	0.47	0.47	0.10 [0.09–0.11]	0.11 [0.10–0.12]	0.33	0.19	0.20
	Alpha 1	0.24 [0.23–0.24]	0.24 [0.23–0.25]	0.30	0.23	0.31	0.16 [0.16–0.17]	0.18 [0.18–0.19]	0.63	0.01	0.08	0.12 [0.11–0.12]	0.12 [0.12–0.13]	0.37	0.14	0.16
	Alpha 2	0.24 [0.23–0.25]	0.24 [0.24–0.26]	0.33	0.19	0.29	0.17 [0.16–0.18]	0.19 [0.18–0.19]	0.49	0.05	0.09	0.12 [0.12–0.13]	0.14 [0.13–0.14]	0.56	0.02	0.04
	Beta 1	0.22 [0.21–0.23]	0.23 [0.22–0.23]	0.47	0.06	0.25	0.16 [0.15–0.17]	0.17 [0.16–0.18]	0.35	0.16	0.17	0.12 [0.10–0.12]	0.13 [0.12–0.13]	0.61	**0.01**	**0.02**
	Beta 2	0.21 [0.20–0.21]	0.22 [0.21–0.23]	0.47	0.06	0.25	0.15 [0.13–0.15]	0.16 [0.15–0.16]	0.54	**0.03**	0.08	0.10 [0.09–0.11]	0.12 [0.12–0.12]	0.75	**< 0.001**	**0.02**
Global efficiency	Delta	0.20 [0.19–0.22]	0.22 [0.21–0.24]	0.42	0.09	0.25	0.17 [0.15–0.18]	0.20 [0.18–0.21]	0.37	0.14	0.15	0.14 [0.12–0.16]	0.16 [0.13–0.17]	0.30	0.23	0.23
	Theta	0.19 [0.17–0.23]	0.21 [0.20–0.23]	0.28	0.27	0.34	0.15 [0.14–0.17]	0.18 [0.17–0.19]	0.47	0.06	0.10	0.12 [0.11–0.13]	0.15 [0.14–0.15]	0.61	**0.01**	**0.02**
	Alpha 1	0.25 [0.23–0.26]	0.27 [0.24–0.35]	0.40	0.11	0.25	0.20 [0.18–0.22]	0.24 [0.22–0.29]	0.56	**0.02**	0.08	0.16 [0.15–0.17]	0.19 [0.18–0.20]	0.68	**0.01**	**0.02**
	Alpha 2	0.31 [0.29–0.32]	0.33 [0.30–0.35]	0.33	0.19	0.29	0.25 [0.24–0.26]	0.29 [0.27–0.31]	0.56	**0.02**	0.08	0.21 [0.20–0.21]	0.23 [0.22–0.24]	0.63	**0.01**	**0.02**
	Beta 1	0.17 [0.16–0.18]	0.17 [0.17–0.18]	0.21	0.41	0.44	0.15 [0.14–0.15]	0.16 [0.15–0.17]	0.40	0.11	0.15	0.11 [0.11–0.12]	0.13 [0.13–0.14]	0.70	**< 0.001**	**0.02**
	Beta 2	0.06 [0.06–0.08]	0.08 [0.08–0.09]	0.51	**0.04**	0.23	0.07 [0.06–0.07]	0.08 [0.07–0.10]	0.44	0.08	0.10	0.05 [0.04–0.06]	0.06 [0.06–0.07]	0.65	**0.01**	**0.02**
Mean nodal strength	Delta	13.76 [13.25–15.41]	15.43 [14.36–16.72]	0.37	0.14	0.25	10.33 [8.71–11.07]	12.58 [10.95–13.47]	0.37	0.14	0.15	7.76 [6.76–9.05]	9.05 [7.39–10.17]	0.35	0.16	0.18
	Theta	13.46 [11.99–15.75]	14.27 [13.55–16.36]	0.26	0.31	0.36	9.26 [8.54–10.23]	11.44 [10.50–12.35]	0.44	0.08	0.10	7.13 [6.21–7.64]	8.57 [8.29–8.69]	0.63	**0.01**	**0.02**
	Alpha 1	17.61 [15.86–18.13]	19.24 [16.47–25.10]	0.37	0.14	0.25	13.01 [11.21–14.16]	15.58 [14.50–18.95]	0.58	**0.02**	0.08	9.48 [9.11–10.14]	10.98 [10.79–12.39]	0.65	**0.01**	**0.02**
	Alpha 2	21.51 [20.26–22.17]	23.52 [21.05–24.62]	0.35	0.16	0.29	15.93 [15.08–17.03]	18.74 [17.47–20.48]	0.58	**0.02**	0.08	12.44 [12.19–13.00]	14.17 [13.39–14.74]	0.61	**0.01**	**0.02**
	Beta 1	11.33 [10.87–12.47]	12.01 [11.76–12.39]	0.23	0.36	0.40	9.24 [8.89–9.70]	10.33 [9.71–11.45]	0.47	0.06	0.10	6.73 [6.51–7.16]	7.98 [7.62–8.34]	0.68	**0.01**	**0.02**
	Beta 2	4.11 [3.71–5.19]	5.39 [5.19–6.58]	0.51	**0.04**	0.23	4.17 [3.90–4.52]	4.76 [4.46–6.30]	0.49	0.05	0.09	2.82 [2.36–3.33]	3.68 [3.48–3.96]	0.72	**< 0.001**	**0.02**
Transitivity	Delta	0.15 [0.14–0.17]	0.17 [0.16–0.18]	0.37	0.14	0.25	0.11 [0.09–0.12]	0.13 [0.11–0.14]	0.37	0.14	0.15	0.07 [0.06–0.09]	0.09 [0.07–0.10]	0.37	0.14	0.16
	Theta	0.15 [0.13–0.17]	0.16 [0.15–0.18]	0.26	0.31	0.36	0.10 [0.09–0.11]	0.12 [0.11–0.13]	0.44	0.08	0.10	0.07 [0.06–0.08]	0.09 [0.08–0.09]	0.63	**0.01**	**0.02**
	Alpha 1	0.19 [0.18–0.20]	0.21 [0.18–0.28]	0.37	0.14	0.25	0.14 [0.12–0.15]	0.17 [0.16–0.20]	0.58	**0.02**	0.08	0.10 [0.09–0.10]	0.11 [0.11–0.13]	0.63	**0.01**	**0.02**
	Alpha 2	0.24 [0.22–0.24]	0.26 [0.23–0.27]	0.37	0.14	0.25	0.17 [0.16–0.18]	0.20 [0.19–0.22]	0.58	**0.02**	0.08	0.13 [0.12–0.13]	0.15 [0.14–0.15]	0.61	**0.01**	**0.02**
	Beta 1	0.12 [0.12–0.14]	0.13 [0.13–0.14]	0.28	0.27	0.34	0.10 [0.09–0.10]	0.11 [0.10–0.12]	0.49	0.05	0.09	0.07 [0.07–0.07]	0.08 [0.08–0.09]	0.68	**0.01**	**0.02**
	Beta 2	0.04 [0.04–0.06]	0.06 [0.06–0.07]	0.51	**0.04**	0.23	0.04 [0.04–0.05]	0.05 [0.05–0.07]	0.51	**0.04**	0.09	0.03 [0.02–0.03]	0.04 [0.04–0.04]	0.75	**< 0.001**	**0.02**

Group 1: MoCA < 24; Group 2: MoCA ≥24. Values for Group 1 and Group 2 are reported as median [IQR]. ES indicates rank-based effect size *r*. *p*FDR indicates Benjamini–Hochberg false discovery rate adjusted *p*-values. Bold values indicate *p*-values or *p*FDR values < 0.05.

#### 3.3.1 18-channel configuration

At the 18-channel level, between-group differences were generally limited and did not survive correction for multiple comparisons (Table 3; Figure 3A). In the alpha 2 band, characteristic path length was higher in group 1 compared to group 2 (*p* = 0.04, *r* = 0.51). In the beta 2 band, several metrics showed group differences, including characteristic path length (*p* = 0.02, *r* = 0.56), global efficiency (*p* = 0.04, *r* = 0.51), mean nodal strength (*p* = 0.04, *r* = 0.51), and transitivity (*p* = 0.04, *r* = 0.51), all with moderate effect sizes. Differences in delta, theta, alpha 1 and beta 1 bands were small and not statistically significant.

#### 3.3.2 64-channel configuration

At the 64-channel level, group differences appeared more pronounced, particularly in the alpha and beta frequency ranges (Table 3; Figure 3B), although they did not survive correction for multiple comparisons. In the alpha 1 band, characteristic path length was higher in group 1 than in group 2 (*p* = 0.01, *r* = 0.65), whereas mean clustering coefficient (*p* = 0.01, *r* = 0.63), global efficiency (*p* = 0.02, *r* = 0.56), mean nodal strength (*p* = 0.02, *r* = 0.58), and transitivity (*p* = 0.02, *r* = 0.58) were higher in group 2. A similar pattern was observed in the alpha 2 band, where characteristic path length remained higher in group 1 (*p* = 0.01, *r* = 0.61), while global efficiency (*p* = 0.02, *r* = 0.56), mean nodal strength (*p* = 0.02, *r* = 0.58), and transitivity (*p* = 0.02, *r* = 0.58) were higher in group 2; mean clustering coefficient showed a similar direction of effect but did not reach conventional significance (*p* = 0.05, *r* = 0.49). In the beta 2 band, characteristic path length was again higher in group 1 (*p* = 0.03, *r* = 0.54), whereas mean clustering coefficient was higher in group 2 (*p* = 0.03, *r* = 0.54); mean nodal strength (*p* = 0.05, *r* = 0.49) and transitivity (*p* = 0.04, *r* = 0.51) showed comparable effects.

#### 3.3.3 173-channel configuration

At the 173-channel level, group differences were stronger and more consistent, with several metrics remaining significant after FDR correction (Table 3; Figure 3C). In the theta band, global efficiency (*p* = 0.01, *p*_FDR_ = 0.02, *r* = 0.61), mean nodal strength (*p* = 0.01, *p*_FDR_ = 0.02, *r* = 0.63), and transitivity (*p* = 0.01, *p*_FDR_ = 0.02, *r* = 0.63) were significantly higher in group 2. In the alpha 1 band, significant differences after FDR correction were observed for global efficiency (*p* = 0.01, *p*_FDR_ = 0.02, *r* = 0.68), mean nodal strength (*p* = 0.01, *p*_FDR_ = 0.02, *r* = 0.65), and transitivity (*p* = 0.01, *p*_FDR_ = 0.02, *r* = 0.63), whereas characteristic path length showed borderline significance after correction (*p* = 0.03, *p*_FDR_ = 0.05, *r* = 0.54). The alpha 2 band showed a similar pattern, with results surviving FDR correction for mean clustering coefficient (*p* = 0.02, *p*_FDR_ = 0.04, *r* = 0.56), global efficiency (*p* = 0.01, *p*_FDR_ = 0.02, *r* = 0.63), mean nodal strength (*p* = 0.01, *p*_FDR_ = 0.02, *r* = 0.61), and transitivity (*p* = 0.01, *p*_FDR_ = 0.02, *r* = 0.61). The largest and most consistent effects were observed in the beta bands, particularly beta 2, where all metrics remained significant after FDR correction, including characteristic path length (*p* < 0.001, *p*_FDR_ = 0.02, *r* = 0.70), mean clustering coefficient (*p* < 0.001, *p*_FDR_ = 0.02, *r* = 0.75), global efficiency (*p* = 0.01, *p*_FDR_ = 0.02, *r* = 0.65), mean nodal strength (*p* < 0.001, *p*_FDR_ = 0.02, *r* = 0.72), and transitivity (*p* < 0.001, *p*_FDR_ = 0.02, *r* = 0.75). A similar pattern was observed in the beta 1 band, where mean clustering coefficient (*p* = 0.01, *p*_FDR_ = 0.02, *r* = 0.61), global efficiency (*p* < 0.001, *p*_FDR_ = 0.02, *r* = 0.70), mean nodal strength (*p* = 0.01, *p*_FDR_ = 0.02, *r* = 0.68), and transitivity (*p* = 0.01, *p*_FDR_ = 0.02, *r* = 0.68) remained significant, while characteristic path length showed borderline significance after correction (*p* = 0.04, *p*_FDR_ = 0.05, *r* = 0.51).

### 3.4 Classification performance

Single-feature binary classification was performed to assess whether individual EEG graph metrics were able to discriminate subjects with lower cognitive performance (group 1) from those with relatively preserved cognition (group 2). Table 4 reports balanced accuracy and AUC values for each combination of electrode configuration, graph-theoretical metric, and frequency band.

**Table 4:** Balanced accuracy and AUC across frequency bands, graph-theoretical metrics, and electrode configurations.

Metric	Band	18 ch.		64 ch.		173 ch.	
		Bal. Acc.	AUC	Bal. Acc.	AUC	Bal. Acc.	AUC
Global efficiency	Alpha 1	0.59	0.57	0.76	0.76	0.76	0.85
	Alpha 2	0.53	0.60	0.71	0.78	**0.76**	**0.86**
	Beta 1	0.41	0.44	0.59	0.63	**0.76**	**0.86**
	Beta 2	0.59	0.71	0.65	0.63	0.70	0.82
	Delta	0.65	0.58	0.65	0.63	0.58	0.57
	Theta	0.64	0.46	0.70	0.65	0.76	0.76
Characteristic path length	Alpha 1	0.53	0.58	0.76	0.79	0.51	0.64
	Alpha 2	0.59	0.63	0.76	0.76	0.51	0.68
	Beta 1	0.52	0.58	0.71	0.68	0.70	0.79
	Beta 2	0.70	0.76	0.65	0.71	**0.76**	**0.86**
	Delta	0.64	0.60	0.58	0.63	0.58	0.58
	Theta	0.64	0.60	0.69	0.63	0.39	0.14
Mean clustering coefficient	Alpha 1	0.59	0.63	0.70	0.76	0.53	0.63
	Alpha 2	0.53	0.53	0.59	0.74	0.70	0.75
	Beta 1	0.65	0.74	0.65	0.58	0.70	0.79
	Beta 2	0.59	0.68	0.70	0.75	**0.76**	**0.94**
	Delta	0.28	0.00	0.52	0.53	0.58	0.54
	Theta	0.11	0.00	0.47	0.44	0.52	0.57
Transitivity	Alpha 1	0.59	0.57	0.70	0.79	0.76	0.85
	Alpha 2	0.53	0.58	0.71	0.78	0.70	0.83
	Beta 1	0.59	0.53	0.65	0.68	**0.82**	**0.89**
	Beta 2	0.59	0.74	0.59	0.67	0.76	0.89
	Delta	0.58	0.53	0.58	0.61	0.58	0.60
	Theta	0.47	0.43	0.70	0.67	0.76	0.76
Mean nodal strength	Alpha 1	0.59	0.56	0.70	0.78	0.82	0.85
	Alpha 2	0.53	0.58	0.71	0.78	0.70	0.82
	Beta 1	0.41	0.47	0.65	0.67	**0.82**	**0.89**
	Beta 2	0.59	0.72	0.65	0.67	0.70	0.88
	Delta	0.58	0.54	0.58	0.61	0.58	0.58
	Theta	0.47	0.43	0.70	0.67	0.76	0.76

For each graph-theoretical metric and frequency band, balanced accuracy (Bal. Acc.) and area under the receiver operating characteristic curve (AUC) are reported separately for the 18-, 64-, and 173-channel configurations. Bold values indicate, across each graph-theoretical metric and electrode configuration, the frequency band associated with the highest balanced accuracy; when balanced accuracy was identical across bands, the band with the highest AUC was highlighted. Higher values indicate better classification performance.

Overall, within the present sample, the strongest classification performance was observed for the 173-channel configuration, which showed the highest balanced accuracy values across graph-theoretical measures. In this configuration, both transitivity and mean nodal strength in the beta 1 band achieved the best overall performance, with a balanced accuracy of 0.82, an AUC of 0.89, a sensitivity of 0.75, and a specificity of 0.89. global efficiency and characteristic path length in the 173-channel montage were associated with balanced accuracy values of 0.76 and AUC values of 0.86, with the best performing bands being alpha 2 and beta 2, respectively. Mean clustering coefficient in the beta 2 band showed the highest AUC overall (0.94), although with a slightly lower balanced accuracy of 0.76.

Within the 64-channel configuration, the best performing models were mainly concentrated in the alpha band. global efficiency and characteristic path length in the alpha 1 band both reached a balanced accuracy of 0.76, with characteristic path length showing the highest AUC in this configuration (0.79). Transitivity and mean nodal strength in the alpha 2 band showed slightly lower but still comparable performance, both with a balanced accuracy of 0.71 and an AUC of 0.78. In contrast, classification performance in the 18-channel configuration was lower overall, with the best result observed for characteristic path length in the beta 2 band, which reached a balanced accuracy of 0.70 and an AUC of 0.76.

**Figure 4:**
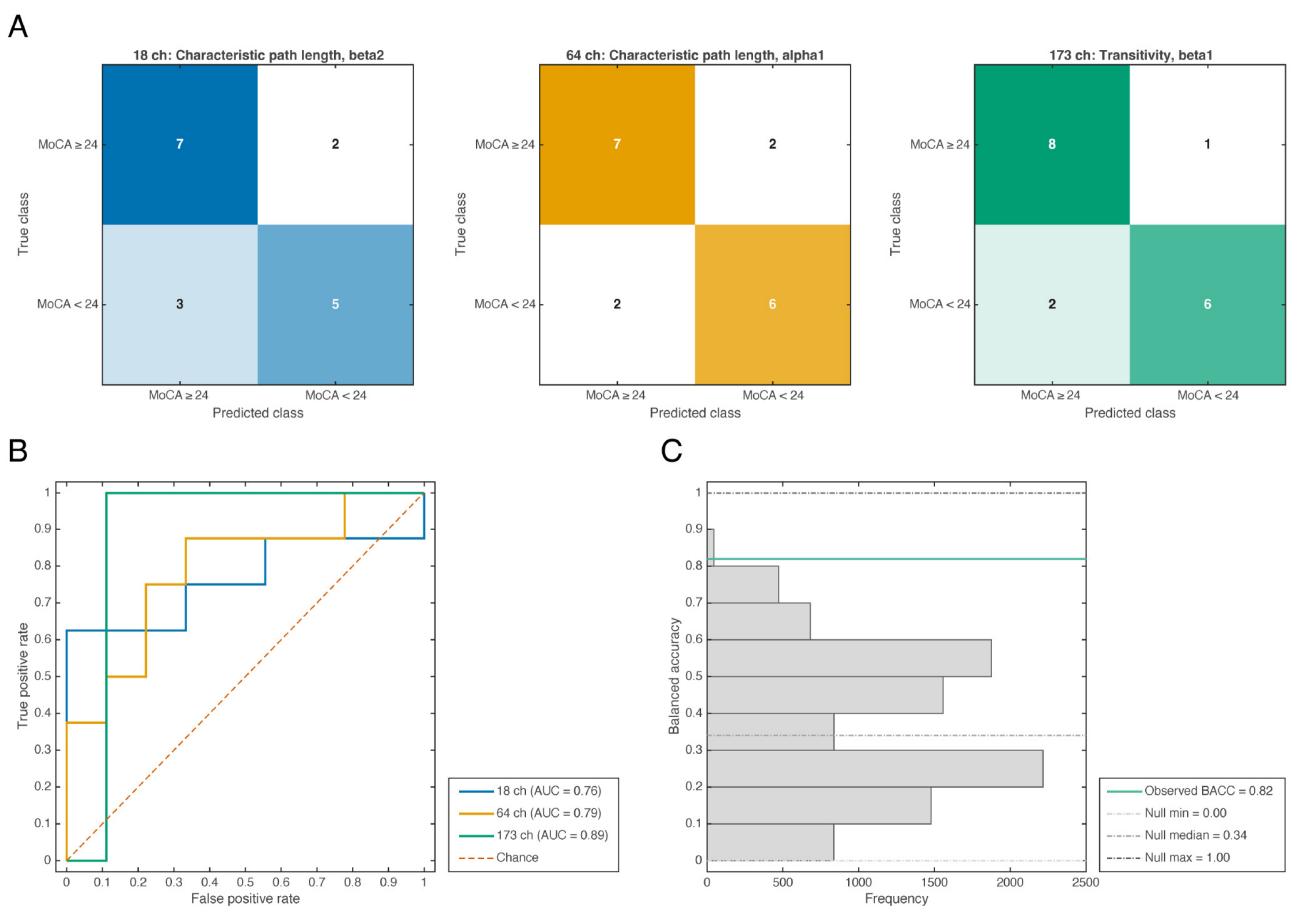
Classification performance of the best single-feature models across electrode configurations. Panel **(A)** Confusion matrices for the best-performing model identified in each electrode configuration: characteristic path length in the beta 2 band for the 18-channel configuration, characteristic path length in the alpha 1 band for the 64-channel configuration, and transitivity in the beta 1 band for the 173-channel configuration. Panel **(B)** Receiver operating characteristic (ROC) curves for the same three models. Panel **(C)** Empirical null distribution of balanced accuracy for the 173-channel transitivity model in the beta 1 band, obtained by permutation testing; the solid horizontal line indicates the observed balanced accuracy, whereas dashed lines indicate the minimum, median, and maximum values of the null distribution. MoCA, Montreal cognitive assessment; AUC, area under the curve; BACC, balanced accuracy.

Figure 4 reports, for the best-performing model identified within each electrode configuration, the corresponding confusion matrices and ROC curves. The confusion matrices highlight the threshold-based classification results for the 18-channel characteristic path length model in the beta 2 band, the 64-channel characteristic path length model in the alpha 1 band, and the 173-channel transitivity model in the beta 1 band. The ROC curves provide a complementary threshold-independent representation of discrimination performance across the three configurations. In addition, for the overall best-performing model, namely transitivity in the beta 1 band with 173 channels, the empirical null distribution of balanced accuracy obtained by permutation testing is shown. This model reached a permutation-based *p*-value of 0.005, indicating that its observed balanced accuracy was unlikely under the null distribution generated by class-label reassignment.

Sensitivity analyses using different MoCA thresholds showed variation in classification performance across cutoffs. Using the threshold at MoCA < 23 (seven subjects with MoCA < 23 and 10 subjects with MoCA ≥23), the best performance was observed for the 173-channel transitivity model in the beta1 band, with an accuracy of 0.82, an AUC of 0.90, and a balanced accuracy of 0.81. The corresponding permutation-based p-value was 0.004. When the threshold was set at MoCA < 25 (11 subjects with MoCA < 25 and six subjects with MoCA ≥25), the best performance was again observed for the 173-channel transitivity model in the beta1 band, although with lower values overall (accuracy = 0.65, AUC = 0.67, balanced accuracy = 0.58), and the corresponding permutation-based *p*-value was 0.086.

## 4 Discussion

The present study investigated whether EEG source-level connectivity and graph-theoretical metrics may be related to global cognitive status in individuals with MCI, and whether electrode density may influence the ability of these measures to reflect and classify cognitive impairment. Two main findings emerged.

First, the variation in correlation strength across electrode configurations may reflect the impact of channel density on the reconstruction of functional brain networks. Low-density EEG provides a relatively coarse representation of connectivity patterns and may therefore be less sensitive to subtle inter-individual differences in network topology associated with cognitive status (Nguyen-Danse et al., 2021; Hatlestad-Hall et al., 2023). By contrast, higher-density configurations offer a more refined spatial sampling of large-scale brain activity, which may improve the estimation of graph-theoretical metrics and enhance the detectability of cognition-related associations (Allouch et al., 2023; Marino and Mantini, 2026). In this context, the emergence of clearer trend-level correlation patterns in the 64- and 173-channel configurations, particularly within the alpha and beta bands, suggests that denser EEG montages may be more sensitive to network properties potentially related to global cognitive functioning. Notably, higher MoCA scores tended to be associated with lower characteristic path length and higher mean clustering coefficient, global efficiency, mean nodal strength, and transitivity, a pattern broadly consistent with a more efficient and integrated network organization supporting better cognitive performance. The predominance of trend-level associations in the alpha and beta ranges is also neurophysiologically plausible, as these frequency bands are closely involved in large-scale cortical communication and higher-order cognitive processes, including attentional control, executive functioning, and information integration (Chapeton et al., 2019). However, none of these associations survived correction for multiple comparisons. Accordingly, these observations should be interpreted cautiously and regarded as preliminary and hypothesis-generating rather than definitive.

Second, the group-comparison and classification analyses showed that the 173-channel configuration revealed the most evident between-group differences, with several metrics remaining significant after FDR correction, particularly in the theta, alpha, and beta bands. Likewise, classification performance improved with increasing channel density: the 173-channel configuration consistently achieved the highest balanced accuracy and AUC values, whereas the 18-channel configuration performed worst and the 64-channel configuration showed intermediate performance. In this context, HD-EEG may provide richer network information and, in the present sample, showed a better discriminative trend. This pattern is consistent with the possibility that HD-EEG captures network alterations associated with relative cognitive status in the present cohort , allowing a clearer separation between individuals with relatively lower and higher global cognitive performance even within a clinically heterogeneous cohort in the MCI spectrum (Cecchetti et al., 2021; Rossini et al., 2020). Importantly, the between-group findings were not limited to a single metric, but instead involved a convergent set of graph properties, including clustering, efficiency, nodal strength, and transitivity, particularly in the higher-density configuration. This convergence supports the interpretation that better cognitive performance may be associated with a more preserved and functionally advantageous network organization. In contrast, the weaker and less stable effects observed in the 18-channel configuration may reflect the reduced ability of sparse montages to resolve subtle topological differences (Smit et al., 2021). The intermediate profile observed for the 64-channel montage further supports the idea of a graded effect of electrode density, rather than a simple dichotomy between low- and high-density systems. However, these classification findings should be considered exploratory and should not be interpreted as definitive evidence of generalizability or of superiority of HD-EEG over lower-density configurations.

The spatial and spectral distribution of the observed effects also deserves consideration. The fact that the clearest differences emerged in the alpha and beta bands, and only became significant in the theta range with 173 channels, suggests that cognitive impairment in MCI may preferentially affect oscillatory systems supporting large-scale coordination and information transfer (Kim et al., 2023). In this sense, HD-EEG may be particularly suited to capturing the network-level expression of these alterations. At the same time, the results indicate that even within an MCI cohort, stratification according to MoCA may reveal substantial neurophysiological heterogeneity, supporting the notion that graph-theoretical EEG measures may be sensitive not only to diagnostic category, but also to gradations of cognitive severity within the same clinical condition.

Taken together, these findings have both methodological and clinical implications. From a methodological perspective, they suggest that electrode density is not a trivial acquisition parameter, but a factor that can directly influence the detectability of cognition-related network effects and the performance of classification models (Allouch et al., 2023; Smit et al., 2021). From a clinical perspective, they suggest the potential utility of HD-EEG as a non-invasive tool for identifying network-based markers of cognitive vulnerability in MCI (Formica et al., 2025; Yuan and Zhao, 2025; Meghdadi et al., 2021). Nevertheless, these conclusions should be interpreted in light of the limited sample size and the exploratory nature of this study. Replication in larger cohorts will be necessary to establish the robustness, generalizability, and possible prognostic value of these EEG-derived network markers.

Furthermore, a convergence between the nonparametric group-comparison results and the classification findings emerged. In the 173-channel configuration, the same metrics that showed the clearest between-group differences, particularly transitivity, strength, global efficiency, and clustering in alpha and beta bands, also produced the best discrimination performance. At the same time, the discrepancy between the correlation analysis and the binary classifications suggests that, within this cohort, the relationship between network measures and cognition may be more readily captured as a contrast between relatively lower and higher cognitive performance than as a strictly monotonic continuous association.

The better performance of the 173-channel montage is plausible, since HD-EEG provides denser coverage of the scalp, which reduces spatial undersampling and improves the estimation of source reconstruction (Marino and Mantini, 2026). This aspect is relevant especially when analyses are performed in source space rather than at the sensor level, as in the present study.

A related point is that source reconstruction from the 18-channel montage is intrinsically more underdetermined than from the higher-density configurations. Therefore, the lower performance observed for the 18-channel montage may partly reflect limitations in source localization rather than connectivity estimation alone. This interpretation is consistent with previous evidence indicating that reduced electrode density can substantially affect source reconstruction (Allouch et al., 2023) and, consequently, functional connectivity estimates and graph-theoretical network characterization (Hatlestad-Hall et al., 2023).

In addition, the study reported in Liu et al. (2018) showed that applying eLORETA to HD-EEG improved the mapping of brain networks.

An additional methodological aspect to consider is the choice of the adopted EEG reference. Common average reference was applied separately to each derived montage. Previous studies have shown that reference choice can influence EEG functional connectivity estimates and derived graph-theoretical properties at the scalp level (Chella et al., 2016; Anastasiadou et al., 2019). Therefore, part of the observed differences across electrode densities may also reflect the interaction between spatial sampling and montage-specific rereferencing, in addition to electrode density itself. Although the analyses were conducted in source space using lagged connectivity measures, which are less sensitive to zero-lag contamination, the potential influence of reference choice on network estimates should still be acknowledged.

A further point to consider is the use of a template-based head model for source reconstruction. In the present study, the inverse solution relied on the standard LORETA-KEY implementation based on the MNI152 template. While this choice ensured methodological consistency across participants and electrode configurations, it may have reduced anatomical accuracy and limited the precision of source localization. This issue is particularly relevant in an elderly cohort within the MCI spectrum, in which cortical atrophy and inter-individual anatomical variability may affect source reconstruction. Therefore, part of the variability observed in source-level connectivity and graph-theoretical measures may also reflect the use of a common template head model rather than subject-specific anatomy.

EEG network-based approaches have increasingly been investigated as tools for classifying cognitive impairment in AD and MCI. Systematic reviews have identified resting-state EEG, and particularly connectivity- and graph-based features, as promising candidate markers, while also highlighting substantial methodological heterogeneity across preprocessing, feature extraction, and validation strategies (Cassani et al., 2018; Paitel et al., 2025). Within this framework, several studies have reported meaningful classification performance using EEG network measures, both for distinguishing MCI from healthy controls and for capturing heterogeneity within the MCI spectrum. In particular, graph- and connectivity-informed models have shown good discriminative ability when combined with cognitive measures or applied to subtype classification and biologically defined stratification (Požar et al., 2020; Youssef et al., 2021; Kim et al., 2022).

The present findings should therefore be interpreted as referring to a clinically heterogeneous early cognitive impairment cohort within the MCI spectrum, rather than to a single biologically uniform MCI subtype. Most prior EEG classification studies have focused on distinguishing MCI or AD from healthy controls, or on combining EEG features with cognitive or multimodal information. By contrast, the present work examined classification within an MCI cohort, using MoCA-defined cognitive stratification rather than diagnostic category. The present analysis systematically evaluated the effect of electrode density on source-level network-based classification, an aspect that has received far less attention than classifier architecture or feature engineering. In this context, the finding that the 173-channel configuration consistently outperformed the 18- and 64-channel montages suggests that increasing spatial sampling may enhance the discriminative value of graph-derived EEG metrics. At the same time, comparisons with the literature should remain cautious, because reported performance depends strongly on sample composition, class definition, preprocessing choices, feature dimensionality, and validation strategy. In addition, the correlation findings should be interpreted more conservatively, since none of the observed trend-level associations survived multiple-comparison correction.

Several limitations should be considered when interpreting the present findings. First, the sample size was relatively small, which reduces statistical power and limits the generalizability of the results. This issue is particularly relevant for the correlation analyses, where several moderate associations emerged but did not survive correction for multiple comparisons, and for the classification analysis, where performance estimates may be affected by sample-specific variability. Although LOOCV and permutation testing were adopted to reduce optimistic bias and to provide an internal estimate of model performance in a small-sample setting, these procedures do not replace independent external validation.

Second, the study did not include a cognitively healthy control group. As a result, the analyses were restricted to variability within the MCI spectrum and do not allow conclusions about the diagnostic ability of the proposed EEG-derived network measures to distinguish MCI from normal aging or from more advanced neurodegenerative stages. The MoCA-based grouping adopted here should therefore be interpreted as an operational stratification of relative cognitive performance within a clinically heterogeneous cohort within the MCI spectrum, rather than as a diagnostic classification framework. The sensitivity analysis showed that the main classification pattern was preserved with the adjacent cutoff at 23, whereas performance decreased at 25. This suggests that the overall result was not limited to the cutoff of 24. However, differences across cutoffs should be interpreted cautiously, since modifying the MoCA threshold also changed the class distribution in this small sample, which may have influenced classification performance.

An additional limitation concerns the clinical and biological heterogeneity of the cohort. Although all participants belonged to the MCI spectrum and were assessed in an early clinical phase, the sample was not etiologically homogeneous and included different clinical presentations, including cases with a vascular component. In addition, amyloid- or tau-based biomarkers were not available, MRI was not available for any participant, and a formal subtype classification was not consistently available. Therefore, the present findings should not be interpreted as evidence of a specific underlying neuropathological substrate, and it remains unclear whether the observed EEG network alterations reflect prodromal AD, mixed pathology, vascular cognitive impairment, nonspecific aging-related changes, or other clinical factors. Accordingly, the biological specificity of the findings is limited. At the same time, this does not preclude the primary exploratory methodological aim of the study, which was to compare EEG-derived connectivity and graph-theoretical measures across different electrode-density configurations within the same cohort.

A further limitation concerns the lack of additional clinical, neuropsychological, and longitudinal variables. The study focused on global cognitive status as measured by the MoCA, but did not include more detailed cognitive profiles, or longitudinal outcomes. Consequently, it remains unclear to what extent the observed network alterations are specifically related to particular cognitive domains, disease subtypes, or underlying AD pathology. Likewise, potentially relevant factors such as education, medication status, vascular comorbidities, and MCI subtype were not incorporated into the analyses and may have contributed to inter-individual variability.

Future studies should aim to replicate these findings in larger and clinically better characterized cohorts, including both cognitively healthy controls and individuals across different stages of cognitive decline. Longitudinal designs would be particularly valuable to determine whether the observed source-level network measures can predict progression from MCI to dementia or track cognitive decline over time. It would also be important to integrate EEG network metrics with more comprehensive neuropsychological, clinical, and biomarker data in order to assess their incremental value within multimodal prediction frameworks.

From a methodological perspective, future research should further investigate the effect of electrode density on EEG-based metrics using larger samples and external validation strategies. While the present results suggest that higher-density montages may improve the sensitivity of source-level connectivity and graph measures, it remains to be established whether comparable performance can be achieved with optimized reduced-channel configurations that are more feasible in routine clinical practice. In addition, future work could explore multifeature and multimodal models, provided that adequate sample sizes and validation procedures are available to minimize overfitting and preserve interpretability.

## 5 Conclusions

The present study suggests that source-level EEG functional connectivity and graph-theoretical measures may be associated with cognitive performance in individuals with MCI, and that these associations may be influenced by electrode density. Among the three montages examined, the 173-channel configuration produced the clearest between-group differences and the best single-feature classification performance, particularly in the alpha and beta frequency bands. These findings suggest that denser spatial sampling may improve the ability of EEG-derived network metrics to capture subtle neurophysiological differences associated with relative cognitive status within the MCI spectrum. Further studies in larger, longitudinal, and clinically better characterized cohorts will be necessary to confirm the robustness of these preliminary findings and to clarify their potential translational relevance for cognitive stratification and screening.

## Data Availability

The raw data supporting the conclusions of this article will be made available by the authors, without undue reservation.
