# Supplementary material for: High-density EEG network analysis in MCI: an exploratory study of electrode density and cognitive performance
Source: Front Aging Neurosci. 2026 Jun 10;18:1854225. doi: 10.3389/fnagi.2026.1854225 (PMC13291014; doi:10.3389/fnagi.2026.1854225)
Supplement: Supplementary file 1 [file Data_Sheet_1.pdf]

Supplementary Material

1 SUPPLEMENTARY TABLES

Table S1: Correspondence between the derived 173-, 64-, and 18-channel montages and the original 256-channel HydroCel layout. The 64-channel montage was obtained as a fixed subset of the 173-channel configuration, and the 18-channel montage as a further fixed subset of the 64-channel configuration.

Index in 173-ch montage	Corresponding index in original 256-ch layout	Included in 64-ch montage	Included in 18-ch montage
1	1	-	-
2	2	✓	✓
3	3	-	-
4	4	-	-
5	5	✓	-
6	6	-	-
7	7	-	-
8	8	-	-
9	9	-	-
10	10	✓	-
11	11	-	-
12	12	✓	-
13	13	-	-
14	14	-	-
15	15	✓	-
16	16	-	-
17	17	-	-
18	18	✓	✓
19	19	-	-
20	20	-	-
21	21	✓	✓
22	22	-	-
23	23	-	-
24	24	✓	-
25	25	-	-
26	26	✓	-
27	27	✓	-
28	28	-	-
29	29	✓	-
30	30	-	-
31	31	-	-
32	32	-	-

Continued on next page

Table S1 – continued from previous page

**Index in 173-ch montage**	**Corresponding index in original 256-ch layout**	**Included in 64-ch montage**	**Included in 18-ch montage**
33	33	-	-
34	34	✓	-
35	35	-	-
36	36	✓	✓
37	37	✓	✓
38	38	-	-
39	39	-	-
40	40	-	-
41	41	-	-
42	42	✓	-
43	43	-	-
44	44	✓	-
45	45	-	-
46	46	✓	-
47	47	✓	✓
48	48	✓	-
49	49	✓	-
50	50	-	-
51	51	-	-
52	52	-	-
53	53	-	-
54	54	-	-
55	55	-	-
56	56	-	-
57	57	-	-
58	58	-	-
59	59	✓	✓
60	60	-	-
61	61	-	-
62	62	✓	-
63	63	-	-
64	64	✓	-
65	65	-	-
66	66	✓	-
67	68	✓	-
68	69	✓	✓
69	70	-	-
70	71	-	-
71	72	-	-
72	74	-	-
73	75	-	-

Continued on next page

Table S1 – continued from previous page

Index in 173-ch montage	Corresponding index in original 256-ch layout	Included in 64-ch montage	Included in 18-ch montage
74	76	✓	-
75	77	-	-
76	78	-	-
77	79	✓	-
78	80	-	-
79	81	✓	-
80	83	-	-
81	84	✓	-
82	85	-	-
83	86	✓	-
84	87	✓	✓
85	88	✓	-
86	89	-	-
87	90	-	-
88	95	-	-
89	96	✓	✓
90	97	✓	-
91	98	-	-
92	99	-	-
93	100	-	-
94	101	✓	✓
95	106	✓	-
96	107	-	-
97	108	-	-
98	109	✓	-
99	110	-	-
100	115	-	-
101	116	✓	✓
102	117	-	-
103	118	-	-
104	119	✓	-
105	124	-	-
106	125	-	-
107	126	✓	-
108	127	-	-
109	128	-	-
110	129	-	-
111	130	-	-
112	131	-	-
113	132	-	-
114	137	-	-

Continued on next page

Table S1 – continued from previous page

Index in 173-ch montage	Corresponding index in original 256-ch layout	Included in 64-ch montage	Included in 18-ch montage
115	138	-	-
116	139	-	-
117	140	✓	-
118	141	-	-
119	142	✓	-
120	143	✓	-
121	144	-	-
122	149	-	-
123	150	✓	✓
124	151	-	-
125	152	-	-
126	153	✓	✓
127	154	-	-
128	155	-	-
129	159	-	-
130	160	-	-
131	161	✓	-
132	162	✓	-
133	163	-	-
134	164	✓	-
135	169	✓	-
136	170	✓	✓
137	171	-	-
138	172	✓	-
139	173	-	-
140	178	-	-
141	179	✓	-
142	180	-	-
143	181	-	-
144	182	-	-
145	183	✓	✓
146	184	-	-
147	185	✓	-
148	186	-	-
149	191	-	-
150	192	-	-
151	193	-	-
152	194	✓	-
153	195	-	-
154	196	-	-
155	197	-	-

Continued on next page

Table S1 – continued from previous page

**Index in 173-ch montage**	**Corresponding index in original 256-ch layout**	**Included in 64-ch montage**	**Included in 18-ch montage**
156	198	-	-
157	202	✓	✓
158	203	-	-
159	204	-	-
160	205	-	-
161	206	✓	-
162	207	✓	-
163	210	✓	-
164	211	✓	-
165	212	-	-
166	213	✓	-
167	214	-	-
168	215	-	-
169	220	-	-
170	221	-	-
171	222	✓	-
172	223	-	-
173	224	✓	✓
